# Design of Digital and Intelligent Financial Decision Support System Based on Artificial Intelligence

**DOI:** 10.1155/2022/1962937

**Published:** 2022-06-20

**Authors:** Tiejun Jia, Cheng Wang, Zhiqiang Tian, Bingyin Wang, Feng Tian

**Affiliations:** Shenhua Group Zhungeer Energy CO. LTD., Ordos, Inner Mongolia 017000, China

## Abstract

The quality of financial decision-making is very important to the future development of an enterprise, but it is often affected by the completeness of useful information for decision-making and the subjective factors of decision makers, and is often unstable. With the development of computer technology, the financial decision support system came into being, which improved the quality of financial decision to some extent. However, although the existing financial decision support system has achieved dataization to a certain extent, it still faces problems such as artificial leadership, insufficient intelligence, and poor decision-making efficiency, and cannot fully meet the needs of decision-makers. The explosion of artificial intelligence technology in recent years has provided potential improvements to financial decision support systems. In this article, we conduct a detailed analysis of the deficiencies in the current financial decision support system, build the mechanism and implementation path of the financial decision support system under artificial intelligence, and design a digital and intelligent financial decision support system. At the same time, we apply the proposed financial decision support system to the financial practice of *X* enterprise. Through the questionnaire survey, it is found that through the comprehensive application of artificial intelligence technology, the new system has a higher degree of intelligence than the existing system, and its construction can effectively improve the timeliness and accuracy of financial decision-making, while reducing the cost of financial decision-making. It is conducive to promoting the integration of management accounting and financial accounting.

## 1. Introduction

The quality of financial decision-making is the life source of an enterprise and is of great significance to the sustainable and stable development of the enterprise. For a long time, the construction of a financial decision support system has been the concern of scholars. The financial decision support system realizes scientific and effective financial decision-making by comprehensively managing the upstream and downstream financial information of the enterprise, integrating the actual financial situation of the enterprise in the whole process, and integrating internal and external information [1–3]. In recent years, with the vigorous development of computer technology, the concepts of digitization and intelligence have emerged in the management information system [4], [5]. Through the digitalization and intelligence of the financial decision-making system, a more complete financial operation environment can be created for enterprises, the behavior of financial personnel can be standardized, and the transparency of financial information can be realized, which has become an important trend of enterprise financial management in the future.

The traditional financial decision support system cannot effectively mine the financial information of enterprises, resulting in the inability to distinguish the value of information, including information and usage information. Judging from the decision analysis system and big data technology implemented by some listed companies and Internet companies, the full mining of data value can greatly improve the efficiency and quality of decision-making [6]. However, most of the current financial decision support systems only implement digital information systems. However, the level of intelligence of financial decision support systems is not satisfactory. Zhao et al. pointed out that the current large-scale financial decision support systems in enterprises are insufficient in intelligence. Specifically, most of the decisions are still judged by junior financial personnel, and the intelligent system cannot make judgments adaptively according to the actual situation. Therefore, the intelligent financial decision support system still has great potential [7].

In order to effectively improve the quality of corporate financial decision-making and promote the deep integration of artificial intelligence technology and the financial field, it is the general trend to build a financial decision support system under artificial intelligence. In order to lay a foundation for the construction of the financial decision support system under artificial intelligence in theory, so as to better guide its construction and application in practice, this article conducts in-depth research on its mechanism and implementation path. Based on artificial intelligence technology, this article investigates the application status of the existing financial decision support system and the necessity and feasibility of the elements contained in the mechanism and implementation path of the financial decision support system under artificial intelligence. On this basis, this article constructs the mechanism and implementation path of the financial decision support system under artificial intelligence. Finally, through the case analysis of the A group that applied the system, the results prove the effectiveness of our proposed system.

## 2. Related Work

The study of decision support systems (DSSs) began in the 1970s, when Scott et al. first proposed the term management decision systems and proposed the application of computers in the decision-making process [8]. The development of the decision support system has gone through five stages: the decision support system with the model base system as the core, the intelligent decision support system (IDSS) combining knowledge reasoning and model calculation, the decision support system based on the data warehouse (DW), the decision support system based on the data warehouse (DW), and client/server (C/S) decision support system and web service-based decision support system. Foreign financial decision support systems started in the 1980s. At the beginning of this century, EXCUCON Systems, Capex, and American Airlines developed and put into use a number of financial decision support systems. At the same time, scholars are constantly proposing new design schemes for financial decision support systems, which greatly strengthens its functions and expands its application fields. Eom et al. proposed a multicriteria decision support system based on an objective planning model to meet the needs of global financial planning of multinational companies [9]. Rigopoulos et al. proposed an intelligent module for strengthening group financial decision support systems, and the operation method of this module is based on multicriteria analysis and is dedicated to helping the financial department to make complex group financial decisions [10].

At the same time, with the development of computer and Internet technology, foreign scholars are also deepening the research on new financial decision support systems, and they have put forward suggestions for improving financial decision support systems from the perspective of specific technical applications. Rupnik and Kukkar proposed the application of data mining technology in decision support systems [11]. Tang et al. proposed a financial decision support system based on information entropy and applied fuzzy logic theory, adaptive genetic algorithm, and other technologies. Marcin et al. believe that many factors can assist financial decision-making, including statistical methods, mathematical methods, behavioral methods, artificial intelligence, and sensitivity analysis of expert opinions. But it is very difficult to predict the market due to uncertainty and risk. In order to better solve this problem, they designed a group financial decision support system based on a consensus method. The design was tested by the data of the Warsaw Stock Exchange [12]. Tang and Leung pointed out that uncertainty has a great impact on the decision-making process, but the existing decision support tools are difficult to solve the instability impact of uncertainty on decision-making [13]. Xiao et al. pointed out the problems in the data collection of the existing system. The existing financial decision support system is established and developed on the basis of the traditional accounting information system. The financial and nonfinancial data obtained through the accounting information system are huge in scale, but contain very little information, which seriously affects the efficiency of financial decision-making [14]. In recent years, artificial intelligence technology has been gradually applied to financial decision support systems. However, Frantz et al. pointed out that the operation of expert systems often requires massive data, but for a long time, these data rely on manual input, and the dependence on manual input data has become a bottleneck in artificial intelligence applications [15].

## 3. The Proposed System

### 3.1. Analysis of Digital and Intelligent Financial Decision Support System

**Figure 1:**
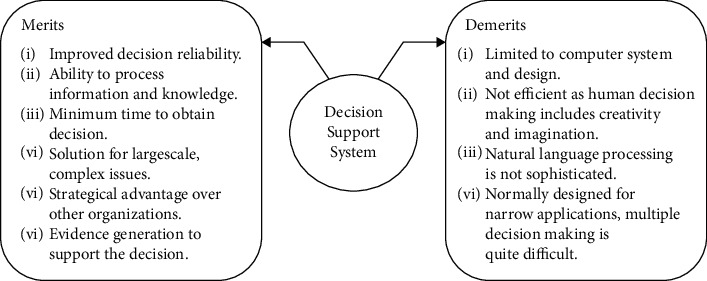
Merits and demerits of the current financial decision support system.

The existing system mainly assists financial decision-making by providing information, which makes it actually mainly play the function of a calculator, and cannot directly provide decision-making suggestions to managers [16–18], as shown in Figure 1. The formulas and decision models stored in the existing system are difficult to update, and the support for financial decision-making is limited to the provision of common financial analysis functions such as DuPont analysis, which makes the output results lack pertinence, especially in the face of unstructured finance. When making decisions, it often fails to provide the analysis results or decision-making solutions that decision makers really need. In addition, as the basis of financial decision-making, the quality of data is very important to the accuracy of financial decision-making, but it is difficult for the existing system to judge the authenticity and reliability of the data collected, which makes decision makers always keep vigilant and guard against data distortion has a limited impact on financial decisions and thus has limited reliance on existing systems.

The financial decision support effect of the existing system is not good [19–21]. In terms of useful information for decision-making, the existing system focuses on the collection of internal business and financial information of the enterprise, while ignoring the collection of external information such as industry information, policy information, and macroeconomic information, and even some key decision-making useful information needs to be supplemented by decision makers, which leads to insufficient decision-making useful information integrity and reduces the quality of decision-making useful information [22–25]. The low quality of useful information for decision-making directly affects the quality of financial decision-making. In addition, for some large group companies, the subordinate companies have their own financial software, the data format is difficult to unify, and the real-time collection and aggregation cannot be realized, which makes these subordinate companies become information islands and increases the difficulty of financial decision-making at the group level. Because the support effect and versatility of the existing systems cannot be achieved at the same time, in order to ensure the quality of decision-making, most of the existing systems are designed for a certain kind of financial decision-making, and it is difficult to solve other financial decision-making problems. At the same time, the construction cost of the financial decision support system is usually high, and it is impossible to build a corresponding system for all problems, so the decision support cost is high and the scope of application is small.

### 3.2. Design of Our Proposed Structure

**Figure 2:**
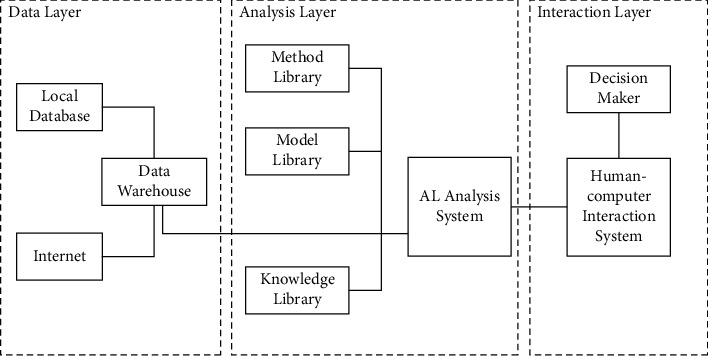
Overall structure of our proposed system.

Overall structure of our proposed system is shown in Figure 2.

The data layer mainly performs data collection, cleaning, data mining, and storage. With the help of automatic data transmission programs and natural language processing technology, it is possible to quickly obtain useful information for internal decision-making such as business and financial information, audit information, and credit information stored in the local database, as well as government policy information, tax information, exchange rate information, and market information published on the Internet information, legal information, macroeconomic information, and other external information. These massive heterogeneous data are cleaned and mined to form multidimensional useful information for decision-making, and are classified and stored in the data warehouse. The data warehouse lays a strong data foundation for the deep learning of the new system and financial decision-making. At the same time, the advance processing and classification of data also provides a guarantee for the timeliness of financial decision-making.

The analysis layer is responsible for carrying out financial analysis, financial forecasting, and financial decision-making activities. Financial analysis is the basis for carrying out financial forecasting and decision-making, and financial decision-making depends on the results of financial analysis and financial forecasting. The analysis layer includes knowledge base, method base, model base, and their respective management systems and artificial intelligence analysis system. The knowledge base stores various financial knowledge, common sense and reasoning rules, and other data, the method base stores financial analysis, forecasting, and decision-making methods, and the model base stores financial analysis models. On the one hand, the management systems of the three databases are responsible for receiving the instructions of the artificial intelligence analysis system and fetching the required knowledge, methods, and models from the corresponding libraries, and on the other hand, they embed deep learning algorithms to automatically carry out new knowledge, new methods, and models in the background. The establishment of new models and the improvement of existing knowledge, methods, and models, so as to update the knowledge base, method base, and model base in time. The artificial intelligence analysis system is responsible for receiving the financial decision-making goals conveyed by the human-computer interaction system and accordingly sends instructions to the database management systems and data warehouses, receives data for analysis, and finally feeds the results back to the human-computer interaction system. The artificial intelligence analysis system includes several inference engines embedded with deep learning algorithms. Some of these inference engines are responsible for determining the types of knowledge, methods, models, and portraits required according to financial decision-making goals, and the other is responsible for financial analysis to generate various portraits. A part is responsible for the generation of financial forecasts and decisions.

The interaction layer is the link between the new system and decision-makers. Human-computer interaction systems use speech recognition and natural language processing technology, so decision-makers can use natural language to communicate with new systems. In the process of financial decision-making, the human-computer interaction system forms financial decision-making goals through the processing of natural language and at the same time communicates the financial decision-making goals to the artificial intelligence analysis system. After completing the financial decision, through the human-computer interaction system, output the financial analysis report, the financial forecast report and the financial decision report that integrates the above report information, or the customized report prepared according to the needs of the decision-maker.

### 3.3. The Digital and Intelligent Mechanism

With the help of the Internet, the new system can obtain massive amounts of structured, semi-structured, and unstructured data such as financial statement information, supply chain information, market information, industry information, securities market information, and online public opinion information in real time. These raw data describe the company's own financial situation and the external financial decision-making environment it faces from many aspects, but these data are chaotic in structure and uneven in quality, and cannot be directly used for financial analysis, so data cleaning and data mining are required. The raw data processed by big data technology have become multidimensional decision-making useful information and is classified and stored by subject. Taking a certain type of product as an example, through multidimensional decision-making useful information, we can extract relevant information from multiple dimensions such as product model, output, sales volume, and main market and quickly obtain the sales of the product at a certain time and place. Just as the liquidity of assets can be judged by indicators such as current ratio and quick ratio, and through these multidimensional decision-making useful information, deep learning algorithms will determine the solvency, profitability, operating ability, growth ability, risk tolerance, risk appetite, and other factors should be evaluated and judged. Compared with the previous evaluation results obtained by solidified indicators, the results obtained by artificial intelligence technology based on exponential indicators are more accurate, thus ensuring the appropriateness of financial decisions. Further carry out financial analysis, financial forecasting, and financial decision-making based on useful information for decision-making. With the help of existing financial analysis methods and corresponding deep learning algorithms, it is possible to analyze and evaluate the solvency, development, profitability, and operational capabilities of enterprises. The financial analysis data together with the enterprise characteristic data constitute the enterprise portrait.

Similarly, through the analysis and processing of useful information for multidimensional decision-making on other topics, various types of financial decision-making information groups such as external environment portraits, asset portraits, and customer portraits can be obtained. When the financial decision target is generated, according to the model obtained by the deep learning algorithm during training, the new portrait is customized, and the various portraits are matched with each other and the future under different matching paths.

Predicting and analyzing the results of financial activities is to obtain financial forecast data. On this basis, the action path that can meet the financial decision-making objective to the greatest extent is selected as the financial decision. Decision-makers can revise the output financial decision through man-machine dialogue, and the revision process will affect the final decision model to improve the quality of the next decision. After obtaining a satisfactory financial decision, decision-makers can choose to output a general financial decision report or customize a personalized report. Useful information for decision-making is the starting point for financial decision-making. Therefore, the new system does not screen information when collecting information, so as to ensure the comprehensiveness of useful information for decision-making. Further processing of these data is required to improve the relevance and availability of useful information for decision-making. For unstructured data, it uses natural language processing technology for structured processing, extracts key entity information, and mines the data relationships contained in this information.

Combining data mining with data-cleaned structured data, the new system can obtain high-quality decision-making useful information including the data itself and the complex relationship between the data. Financial decision-making methods and models are the link between useful information for decision-making and financial decision-making, reflecting the logical relationship and causal relationship between the two. Therefore, financial decision-making methods and models have a significant impact on the quality of financial decision-making. Here, we introduce two classical deep learning methods as decision models, namely, recurrent neural network (RNN) and long short-term memory. The equation for RNN is denoted as

(1) $$\begin{array}{c} o_{t}=g\left(W_{o}s_{t}\right),\\ s_{t}=f\left(W_{x}x_{t}+W_{s}s_{t-1}\right). \end{array}$$

For LSTM, the equation is denoted as

(2) $$\begin{array}{c} f_{t}=\sigma \left(W_{f}x_{t}+U_{f}h_{t-1}+b_{f}\right),\\ i_{t}=\sigma \left(W_{i}x_{t}+U_{i}h_{t-1}+b_{i}\right),\\ o_{t}=\sigma \left(W_{o}x_{t}+U_{o}h_{t-1}+b_{o}\right),\\ c_{t}=f_{t}⊙c_{t-1}+i_{t}⊙\sigma \left(W_{c}x_{t}+U_{c}h_{t-1}+b_{c}\right),\\ h_{t}=o_{t}⊙\sigma \left(c_{t}\right), \end{array}$$

where *x*_*t*_ is the input features, which consist of price features and technical indicator. *f*_*t*_, *i*_*t*_, and *o*_*t*_ represent forget gate, input gate, and output gate, respectively. *c*_*t*_ and *h*_*t*_ represent cell state and hidden state, respectively.

Because our model is deployed on a financial system, computational complexity is a consideration, we improve the LSTM so that it can be deployed on high-traffic financial decision support systems. Specifically, the improved LSTM formula is as follows:

(3) $$\begin{array}{c} z_{t}=\sigma \left(W_{z}\left[h_{t-1},x_{t}\right]\right),\\ r_{t}=\sigma \left(W_{r}\left[h_{t-1},x_{t}\right]\right),\\ \hat{h_{t}}=\tanh\left(W\left[r_{t}h_{t-1},x_{t}\right]\right),\\ h_{t}=\left(1-z_{t}\right)h_{t-1}+z_{t}\hat{h_{t}}. \end{array}$$

Meanwhile, considering the black-box characteristics of the deep learning model, we added attention mechanism in the improved LSTM to improve the interpretability of the model. The attention mechanism enables models to adaptively discover potential patterns in input data, thereby improving model performance by identifying important features. Besides, the identification of important features also provides guidance for financial personnel to explain the decision-making process of the model. The formula for the attention mechanism is as follows:

(4) $$\begin{array}{c} e_{t}=h_{t}Wc_{T},\\ \alpha_{t}=\frac{\exp\left(e_{t}\right)}{\sum_{t=1}^{T}\exp\left(e_{t}\right)},\\ h=\sum_{t=1}^{T}\alpha_{t}e_{t} \end{array}$$

Using deep learning algorithms, we feed the new system with decision-making information, and if it makes the right financial decision, we give the neural network that made the right decision more weight, and less weight if it does not. This process is the training process of the new system. After training enough times, the new system will sum up its own financial decision-making methods and models, making financial decisions without human involvement. These financial decision-making methods and models may be different from the current solidified models. Compared with solidified models, these methods and models are more complex functional systems and have a higher degree of fitting to the data. And as the number of training increases, these methods and models will become more complex, and the quality of financial decisions will continue to improve. When the new system receives the financial decision objective, the financial decision support process is initiated. The new system will select the financial decision-making methods and models that have been trained according to the financial decision-making objectives and select useful information for decision-making according to the methods and models. After calculation and analysis, a financial decision is finally generated.

## 4. Empirical Study

We execute real applications at *X* company and collect data for assessment in order to verify the efficacy of the suggested financial decision support system. Internal demands, rules, objectives, and external pressures are all factors that financial decision support systems consider. As a result, before the update, each category is assessed against the planned structure and compared to Enterprise *X*'s decision support system: *S*1 for internal pressure, *T*1 external pressure, *U*1 goals and financial criteria, with subgoals, JA1 job satisfaction (common to financial employees), JA2 cognitive skills, JA3 employee contribution to the company, JA4 employee strength, JA5 individual effort, JA6 timely reaction, JA7 accessible resources, JA8 organizational characteristics, and JA9 maturity to company activities. The classification results are grouped into *U*1 and *U*2 groups, and the final classification result is generated as an output of the organizational development decision support system.

**Figure 3:**
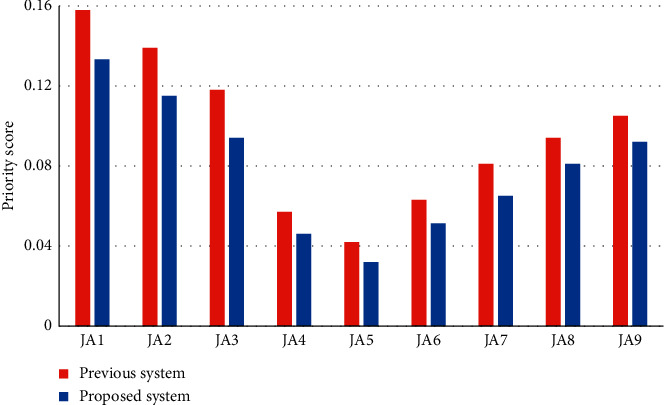
The results of the priority assessment comparison.

The priority function value is obtained based on the consistency of the clusters and thresholds. If the value is below 0.1, it is acceptable, and others are considered unacceptable. Based on the weights, the priority value of the proposed system is measured and compared with the previous system, as shown in Figure 3. By evaluating employees, stack holders, and management, it was observed that the proposed model had better priority value opinions. While the previous system has a lower priority value due to its inefficient evaluation strategy.

The priority values of our proposed system are assessed and compared to the existing system using the weights, as illustrated in Figure 3. It was discovered that the suggested approach had superior priority value opinions after examining workers, stack holders, and management. Other models, on the other hand, have lower priority ratings owing to ineffective assessment techniques.

## 5. Conclusion

The application of the financial decision support system can have a positive impact on enterprise financial decision-making, including making financial analysis more comprehensive and accurate, improving the comprehensiveness of useful information for decision-making, and improving the timeliness of financial decision-making. However, the existing system is not widely used, and its functions are not comprehensive enough to meet all the decision-making needs of decision makers. The main reason for this situation is that the existing systems generally have the problems of low degree of intelligence, high cost of system construction and operation, insufficient timeliness of decision-making support, and poor decision-making effect. By realizing the extensive application of artificial intelligence technology in financial decision support systems, a financial decision support system under artificial intelligence can be constructed. The application of digital and intelligent systems can provide decision-makers with more comprehensive and accurate decision-making useful information, and under the condition of ensuring the principle of cost and benefit, expand the scope of financial decision support, improve the scientificity and objectivity of financial decision-making, and reduce the probability of irrational decision-making. It will improve the overall financial decision-making quality of the enterprise and ensure the long-term stable and healthy development of the enterprise. This article proposes a financial decision support system composed of data layer, analysis layer, and interaction layer. Driven by digitalization and intelligence, it provides financial analysis, forecasting, and decision support services including financing decisions, investment decisions, cost decisions, dividend distribution decisions, and special financial decisions. AI technology is applied throughout the financial decision-making process. First of all, the new system mines and organizes the data collected in each basic database, draws various portraits, and stores them in the data warehouse. When the decision-making target is received, it supplements the decision-making useful information according to the decision-making target, and performs portrait matching, so as to obtain financial decision-making. Subsequently, the scheme can be modified and improved with the help of human-computer interaction to form the final scheme. The empirical research on the actual application of *X* enterprise shows that the system we propose not only inherits the digital characteristics of the existing system but also better utilizes intelligent technology to organize the flow of data, and realizes the improvement of decision-making efficiency.

## Data Availability

The data used to support the findings of this study are available from the corresponding author upon request.
